# Dynamics of Short-Term Phosphorus Uptake by Intact Mycorrhizal and Non-mycorrhizal Maize Plants Grown in a Circulatory Semi-Hydroponic Cultivation System

**DOI:** 10.3389/fpls.2017.01471

**Published:** 2017-08-25

**Authors:** Mónica Garcés-Ruiz, Maryline Calonne-Salmon, Katia Plouznikoff, Coralie Misson, Micaela Navarrete-Mier, Sylvie Cranenbrouck, Stéphane Declerck

**Affiliations:** ^1^Laboratory of Mycology, Earth and Life Institute, Université catholique de Louvain Louvain-la-Neuve, Belgium; ^2^Laboratorio de Micología, Facultad de Ciencias Exactas y Naturales, Pontificia Universidad Católica del Ecuador Quito, Ecuador; ^3^Mycothèque de l’Université catholique de Louvain (BCCM/MUCL), Laboratory of Mycology, Earth and Life Institute, Université catholique de Louvain Louvain-la-Neuve, Belgium

**Keywords:** *Rhizophagus irregularis*, maize (*Zea mays* L.), short-term phosphorus depletion, phosphorus uptake, Hoagland modified solution, non-destructive, circulatory semi-hydroponic cultivation system

## Abstract

A non-destructive cultivation system was developed to study the dynamics of phosphorus (Pi) uptake by mycorrhizal and non-mycorrhizal maize plantlets. The system consisted of a plant container connected via silicon tubes to a glass bottle containing a nutrient solution supplemented with Pi. The nutrient solution is pumped with a peristaltic pump to the upper part of the container via the silicon tubes and the solution percolate through the plantlet container back into the glass bottle. Pi is sampled from the glass bottle at regular intervals and concentration evaluated. Maize plantlets were colonized by the AMF *Rhizophagus irregularis* MUCL 41833 and Pi uptake quantified at fixed intervals (9, 21, and 42 h) from the depletion of the Pi in the nutrient solution flowing through the plantlets containers. Plants and fungus grew well in the perlite substrate. The concentration of Pi in the bottles followed an almost linear decrease over time, demonstrating a depletion of Pi in the circulating solution and a concomitant uptake/immobilization by the plantlet-AMF associates in the containers. The Pi uptake rate was significantly increased in the AMF-colonized plantlets (at 9 and 21 h) as compared to non-colonized plantlets, although no correlation was noticed with plant growth or P accumulation in shoots. The circulatory semi-hydroponic cultivation system developed was adequate for measuring Pi depletion in a nutrient solution and by corollary Pi uptake/immobilization by the plant-AMF associates. The measurements were non-destructive so that the time course of Pi uptake could be monitored without disturbing the growth of the plant and its fungal associate. The system further opens the door to study the dynamics of other micro and macro-nutrients as well as their uptake under stressed growth conditions such as salinity, pollution by hydrocarbon contaminants or potential toxic elements.

## Introduction

The arbuscular mycorrhizal fungi (AMF) are normal and ubiquitous biota belonging to the soil (Smith and Smith, 2010). They occupy a singular place at the interface between soil and roots, colonizing a wide variety of plant species (Smith and Read, 2008), providing minerals to their hosts [mainly phosphorus (P)] in exchange for carbon (Smith and Read, 2008; Smith et al., 2011). These fungi also increase the resistance/tolerance of plants to a/biotic stresses (Smith et al., 2011).

A major benefit of AMF to plants is the increased/facilitated acquisition of Pi via the so-named AMF pathway, thus bypassing direct uptake by roots (Javot et al., 2007), as evidenced in maize (Wright et al., 2005; Tian et al., 2013). The contribution of AMF to Pi uptake and plant nutrition has been investigated extensively (Smith et al., 2011) under greenhouse (Nouri et al., 2014; Walder et al., 2015), in the field (Yang et al., 2014) and for some under *in vitro* culture conditions (Dupré de Boulois et al., 2006; Calonne et al., 2014) using various isotopes (i.e., ^33^P and ^32^P). Studies were almost all destructive (Castillo et al., 2013; Nazeri et al., 2014; Xie et al., 2014; Yang et al., 2014; Gonçalves de Oliveira et al., 2017) thus preventing continuous measurements of Pi uptake by intact non-perturbed plant-AMF associates. Studies conducted by Colpaert et al. (1999) and Van Tichelen et al. (1999) with ectomycorrhizal (ECM) fungi implemented a short-term Pi uptake dynamics on intact ECM and non-mycorrhizal *Pinus sylvestris* seedlings using a non-destructive semi-hydroponic cultivation system (Colpaert et al., 1999). The depletion of Pi was monitored in a nutrient solution percolating through plant containers. The authors concluded that the extraradical mycelium of *Paxillus involutus, Suillus luteus, S. bovinus* and *Thelephora terrestris* strongly influence the Pi-uptake capacity of the pine seedlings. Van Tichelen et al. (1999) used this system to monitor nutrients uptake by the ECM-colonized *P. sylvestris* plantlets under copper toxicity and demonstrated that mycorrhizal plants consistently had higher Pi uptake capacities than non-mycorrhizal plants.

In the present study a circulatory semi-hydroponic cultivation system, derived from the system of Colpaert et al. (1999) for ECM, was used to analyze the short-term Pi uptake dynamics in non-mycorrhizal and AMF-colonized maize plantlets. For the first time, short-term Pi uptake was studied on intact plant–AMF association. The measurements were non-destructive so that the time course of the Pi uptake capacity of individual plants could be followed. Pi depletion in the nutrient solution was monitored during 42 h and converted into Pi uptake rates by the plant/fungus associates and in shoots and roots concentration of P at the end of the experiment.

## Materials and Methods

### Biological Material

The AMF *Rhizophagus irregularis* (Błaszk., Wubet, Renker and Buscot) C. Walker and A. Schüßler as [‘irregulare’] MUCL 41833 was supplied by the Glomeromycota *in vitro* collection (GINCO^1^). The fungus was proliferated on *Zea mays* L. cv. ES Ballade (Euralis, France) in a plastic box containing a sterilized (121°C for 15 min) substrate of vermiculite and sand (w:w, 1:1) supplemented with 20 beads of Osmocote^®^Pro 5-6M (NPK 17-11-10+2MgO+TE). In parallel, *Z. mays* L. seeds were inoculated in a similar substrate without *R. irregularis* (i.e., the non-mycorrhized – NM – control). The AMF and NM control plastic boxes were maintained under greenhouse conditions at 25°C/18°C (day/night), a relative humidity (RH) of 38%, a photoperiod of 16 h day^-1^ and a photosynthetic photon flux (PPF) of 120 μmol m^-2^ s^-1^. Root colonization (see *AMF Root Colonization Determination*) was estimated after 2 months on three randomly selected maize plantlets. The total root colonization reached 87% ± 0.2, while no colonization was noticed for the NM control plantlets. Chopped roots and substrate from both inocula were used in the experiments below.

Maize (cv. ES Ballade) was supplied by the Centre Indépendant de Promotion Fourragère (CIPF^2^). The seeds were surface-disinfected by immersion in sodium hypochlorite (8% active chloride) for 15 min and rinsed three times with sterilized (121°C for 15 min) deionized water during 10 min. The seeds were then placed on wet paper and germinated in plastic boxes in the dark at room temperature (∼20°C). Eighty percent of the seeds germinated within 3 days.

### Short-Term Pi Depletion in a Nutrient Solution Circulating through Containers with Pre-mycorrhized and Non-mycorrhized Plantlets

#### Pre-mycorrhization of Maize Plantlets

For the pre-mycorrhization of maize plantlets, two plastic nursery boxes (Modiform, Netherlands) with compartments of 0.08 L (4 cm × 4 cm × 5 cm size) were used. Thirty grams of lava stone substrate (DCM, Belgium) mixed with 1 g of AMF inoculum was added in each compartment and covered with 33 g of substrate. A control was similarly set up with the NM inoculum. Three-days-old maize seedlings were planted in each box. The seedlings were kept under greenhouse conditions set at 25/20°C (day/night), a RH of 50%, a photoperiod of 16 h day^-1^ and a PPF of 150 μmol m^-2^ s^-1^.

Just before transfer in containers, three randomly selected pre-mycorrhized (PM) plantlets and NM plantlets were harvested and analyzed for root colonization. The total root colonization was 80.7% ± 1.7 for the PM plantlets, while no colonization was noticed for the NM plantlets.

#### Experimental Set up

**FIGURE 1:**
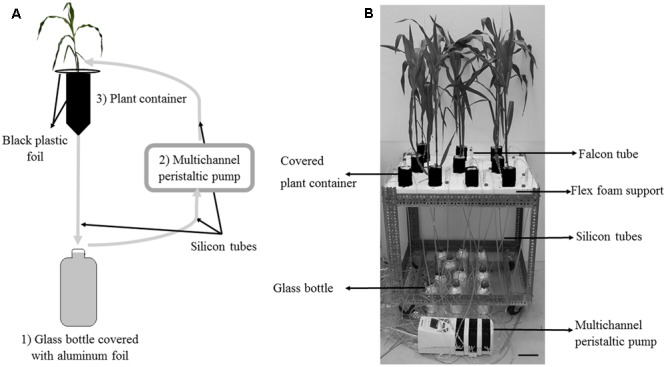
The circulatory semi-hydroponic cultivation system. **(A)** Schematic representation of the experimental set-up to monitor Pi depletion in Hoagland^-P^ solution circulating through containers with pre-mycorrhized (PM) or non-mycorrhized (NM) maize plantlets. The nutrient solution in the glass bottle (1) is pumped with a peristaltic pump (2) to the upper part of the container (3) via silicon tubes. The solution percolate through the plantlet container back into the glass bottle. Gray arrows indicate the direction of flow of the nutrient solution in the tubing. **(B)** Experimental set up under greenhouse conditions. One table contained randomly disposed containers. Scale bar: 10 cm.

After 17 days of pre-mycorrhization, 9 PM maize plantlets and the same number of NM plantlets were transferred into the containers (**Figure 1**) containing dry perlite. The roots of the PM and NM plantlets were rinsed with deionized water to eliminate the lava stone substrate and seed debris prior to transfer into the containers.

Containers consisted of inverted 75 mm diameter wash bottles (500 mL – VWR, United States) cut at the base. A mesh of 100 μm size pore (Prosep B.V.B.A., Belgium) was glued on the top of the bottle to avoid losses of the growing substrate and to prevent roots to grow outside the bottle. Each bottle was filled with 32 g perlite (KNOUF- GMBH, Germany), beforehand sieved at 1 mm diameter, rinsed with deionized water and dried in an oven for 48 h at 50°C. Maize plantlets were kept in the same conditions as above.

According to the equivalence test (Robinson et al., 2005), the height of plantlets presented a difference (*P* = 0.74) between the PM (36.7 ± 1.2 cm) and NM (32.4 ± 3.3 cm) treatments.

The containers with plantlets were randomly disposed in holes made in flex foam supports on 3 separate tables. A control without plantlet was also included (**Figure 1B**). Each container was wrapped with black plastic foil to prevent algae development. The surface of the perlite was covered with the same black plastic foil (**Figure 1B**).

Before set up of the system, the PM and NM maize plantlets were acclimatized during 25 days in the perlite. The plantlets received 200 mL of Hoagland^-P^ solution every 48 h (containers were closed and old solution was discarded before fresh was introduced). The modified Hoagland (Hoagland and Arnon, 1950) solution (i.e., 90% P-impoverished solution – Phosphorus = 6.245 mg L^-1^, referred as Hoagland^-P^ throughout the text) contained in mg L^-1^ deionized water: NH_4_NO_3_, 80; Ca(NO_3_)_2_⋅4H_2_O, 826; KNO_3_, 357; KCl, 45.1; K_2_SO_4_ 105.4; KNO_3_, 50; KH_2_PO_4_, 27.4; MgSO_4_, 120.4; MnSO_4_⋅H_2_O, 0.5; H_3_BO_3_, 1.4; CuSO_4_⋅5H_2_O, 0.2; (NH_4_)_6_Mo7O_2_⋅4H_2_O, 0.1; ZnSO_4_;7H_2_O, 0.6 and Fe-EDTA, 19. The pH of the solution was adjusted to 5.6 ± 0.2 before use.

After acclimatization, Pi depletion in a fresh circulating Hoagland^-P^ solution was monitored in the PM and NM maize plantlets.

#### Monitoring Pi Depletion in Hoagland^-P^ Solution

A circulatory semi-hydroponic cultivation system was set up to monitor the short-term depletion of Pi in the modified Hoagland solution. The Hoagland^-P^ solution circulated via a peristaltic pump through the containers with PM or NM maize plantlets as described by Colpaert et al. (1999), Van Tichelen et al. (1999) and Crahay et al. (2013) for ECM fungi (see detailed description in **Figures 1A,B**). From the Pi depletion in the circulating solution, P uptake rates were deduced for the PM and NM maize plantlets.

The system consisted of 1 L glass bottles covered with aluminum foil and containing the Hoagland^-P^ solution. Sterilized (sodium hypochlorite solution 50% overnight) 3 mm inner diameter silicon tubes (VWR, United States) linked the maize plantlets containers to the glass bottles via a multichannel peristaltic pump (Gilson’s Minipuls Evolution, France). Each plantlet was connected to a single bottle. The tubes plunged in the bottles and the solution was pumped to the containers. The bottom of the containers was similarly connected to another silicon tube and the solution percolated through the plantlets and returned to the individual bottles via a silicon tube (**Figures 1A,B**).

**FIGURE 2:**
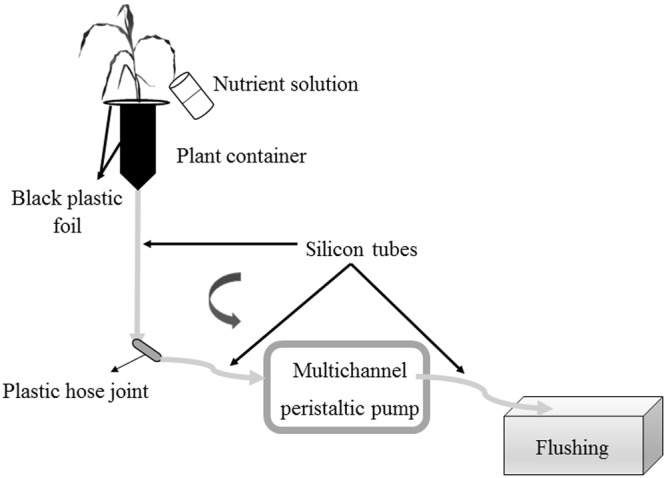
Schematic representation of nutrient solution flushing before Pi short-term monitoring. Hoagland^-P^ solution was added to the plant (4 × 50 mL). Silicon tubes were unified with a plastic hose joint. The multichannel peristaltic pump was turned on. Gray arrows indicate the direction of flow of the nutrient solution in the tubing.

Prior to the Pi-depletion short-term experiment, a flushing was performed to establish an equal nutrient concentration in all containers (Colpaert et al., 1999). Each plantlet received 200 mL (4 aliquots of 50 mL) of Hoagland^-P^ solution. The flow rate in the multichannel peristaltic pumps was 44 mL min^-1^ (**Figure 2**). One L of Hoagland^-P^ solution per plantlet and control containers was prepared. Twenty milliliter of the solution was sampled from each bottle before the start of the circulatory system [considered as Time 0 (T_0_)] to determine initial Pi concentration in the glass bottles. The circulating system was then initiated at a speed of 7.4 mL min^-1^. At defined intervals, the nutrient solution was sampled in Falcon tubes of 50 mL. At each sampling time, the pumps were stopped, the end of the silicon tubes connecting the pump to the plantlets were placed over Falcon tubes and the pumps set on again. Similarly, after sampling, the pumps were stopped, the silicon tubes placed over the containers, and pumps set on again. Twenty milliliter of nutrient solution was collected at 9, 21, and 42 h following the start of the nutrient flow. The samples were then stored at 4°C in the dark before Pi analysis by inductive coupled plasma atomic emission spectrometer (ICP-AES).

#### Maize Harvest and Analysis

At the end of the experiment, i.e., 25 days after transfer into perlite, the plantlets were harvested. The shoot and roots fresh weights (SFW and RFW, respectively) were estimated before drying in an oven at 50°C for 48 h and evaluation of shoot, root and total dry weights (SDW, RDW, and TDW, respectively). The root systems were subsequently separated in two parts to evaluate AMF root colonization and P concentration.

#### AMF Root Colonization Determination

Root colonization was estimated on three replicates from the PM and NM plantlets before experiments set up and all the PM replicates at the end of the experiment (i.e., at 17 and 46 days, respectively). Roots were sampled and analyzed via ink staining (Walker, 2005). The roots were cut into small pieces and placed in Falcon tubes (Sarstedt, Germany). Twenty-five milliliter of KOH 10% was added to the roots before incubation at 70°C in a water bath for 1 h. The KOH was then removed and roots washed with HCl 1%. The staining step consisted in adding 25 mL of ink 2% (Parker blue ink, United States) in HCl 1%. The tubes were then placed at 70°C in a water bath for 1 h. The roots were rinsed and stored in deionized water before observation (Walker, 2005). Total (TC), arbuscular (AC) and vesicular (VC) colonization were estimated under a dissecting microscope (Olympus BH2–RFCA, Japan) at 10× magnification (McGonigle et al., 1990). Around 170 intersections were observed per plantlet. At each intersection the presence or absence of the fungus was noted.

### Phosphorus Quantification

#### Phosphorus Quantification in Hoagland^-P^ Solution

The Hoagland^-P^ solution was sampled at 0, 9, 21, and 42 h and Pi concentration assessed. Two milliliter of each sample was diluted five times with ultrapure water (Millipore, France). The solution was then acidified with 20 μL of HNO_3_ at 65% (Merck, Germany) and subsequently analyzed by ICP-AES (ICAP 6500, Thermo-Scientific, United States). The Pi content was determined with axial viewing of the emitted radiation. A peristaltic pump was used to introduce the solutions into the ICP-AES at a flow rate of 1.5 mL min^-1^. Operating parameters for the instrument included forward power 1150 W, coolant gas flow rate 12 L min^-1^, auxiliary gas flow rate 1 L min^-1^ and nebulizer gas flow rate 0.6 L min^-1^. Pi quantification was analyzed under a wavelength of 177.495 λ nm^-1^. The limit of detection was <100 ppb. Data obtained (in ppm) were converted in mg L^-1^.

The Pi depletion values obtained from the medium were standardized according to those obtained by their respective blanks and Pi concentration at time 0 h, following the formula below:

$$\begin{array}{c} {\left[P\right]}_{X}={\left[P\right]}_{X}\mathrm{quantified}\mathrm{with}ICP-AES\mathrm{at}\mathrm{time}T+\left({\left[P\right]}_{\mathrm{blank}}\mathrm{at}T_{0}-{\left[P\right]}_{\mathrm{blank}}\mathrm{at}T\right) \end{array}$$

where:

[P] = Pi concentration in the solution
X = sample
blank = blank (i.e., perlite control) respective to the sample analyzed
T = time considered (9, 21, or 42 h after the start of the circulatory system)
T_0_ = 0 h before the start of the circulatory system
Net Pi uptake was determined from the depletion of Pi in a Hoagland^-P^ solution circulating through the plantlet containers.

#### Phosphorus Quantification in Maize Plantlets

After evaluation of dry weight, 300 mg of shoot and 100 mg of roots were ground separately in a grinder for P analysis. The ground material was then placed at 50°C overnight. Each sample was transferred into an Erlenmeyer (Pyrex, United States) and 4 mL of HNO_3_ 65% was added for the digestion process. Erlenmeyers were covered with a watch-glass and placed on a heating plate at 60°C overnight. After concentration at 120°C, samples were diluted with 3 mL HCl 38% (Merck, Germany), 1 mL HNO_3_ 65% and 10 mL of ultrapure water (Millipore, France). The solution was filtered with filter paper N°1 (Whatman, United Kingdom) in a 25 mL volumetric flask, before analysis. P concentration was converted from ppm to mg Kg^-1^ and content of P was determined according to the dry weight from shoot part and root system.

### Statistical Analysis

Equivalence test (Robinson et al., 2005) was used to determine differences in height of maize plantlets before transfer into the containers.

Plant dry weight, P concentration and root colonization were analyzed by a student’s *t*-test and the homogeneity of variance was tested (Levene).

Phosphorus depletion in the Hoagland^-P^ solution was firstly subjected to a repeated-measures ANOVA with REML estimation where “Time” (of sampling, i.e.: 0, 9, 21, 42 h) and “table” (3 different flex foam supports in the greenhouse) were regarded as random factors. Each time was secondly analyzed independently using a mixed model, with “AMF” as fixed factor and “table” as random factor.

Normal distribution of residuals was checked and non-normal data were normalized by log transformation before analysis. Results are followed by the standard error (SE). All the tests were performed using the IBM SPSS statistic 23 software and JMP Pro 12 statistical software (SAS Institute Inc., United States).

## Results

### Short-Term Pi Depletion

**FIGURE 3:**
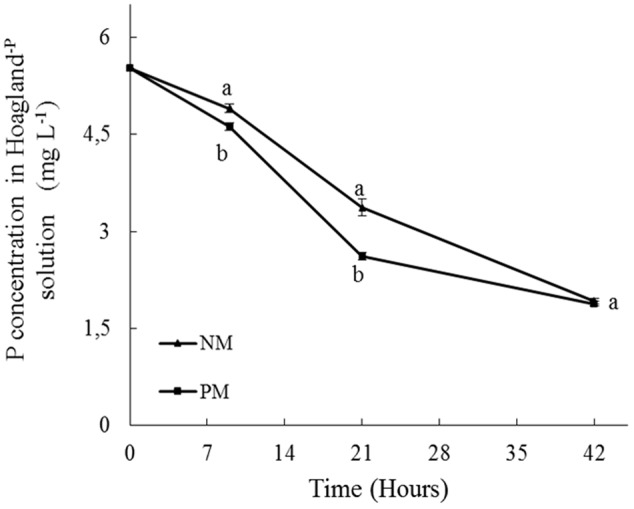
Short-term time course of phosphorus (Pi) depletion (mg L^-1^) from a 90% P-impoverished Hoagland solution (i.e., *P*i = 6.245 mg L^-1^) circulating through plant containers in presence of pre-mycorrhized (PM) and non-mycorrhized (NM) maize plantlets. Pi was sampled at 0, 9, 21, and 42 h of circulating solution. Data are mean values and standard errors (SE) of 8 (NM) and 9 (PM) replicates. Different letters at 9, 21, and 42 h indicate significant differences between treatments.

The concentration of Pi in the bottles of the PM and NM treatments followed the same time-course with an almost linear decrease over time (**Figure 3**), demonstrating a depletion of Pi in the circulating solution and a concomitant uptake/immobilization by the plantlet-AMF associates in the containers. However, differences were noticed between both treatments, as noticed by the repeated-measures ANOVA (*P* = 0.0103, results not shown). A mixed model was thus used to determine the difference in Pi absorption by PM and NM plantlets for each time. At 9 h, the concentration of Pi in the bottles (i.e., the nutrient circulating solution) was significantly higher in the NM treatment as compared to the PM treatment (*P* = 0.037). Consequently, the relative Pi uptake/immobilization in the PM treatment was significantly higher than in the NM treatments (i.e., 16.4% versus 11.3% of the initial Pi concentration in the PM and NM treatments, respectively). A similar observation was made at 21 h. The Pi concentration in the bottles was significantly higher in the NM treatment as compared to the PM treatment (*P* = 0.002) and as a consequence the relative Pi uptake/immobilization in the PM treatment was significantly higher as compared to the NM treatment (i.e., 53 and 38.5% of the initial concentration of Pi in the PM and NM treatments, respectively). Conversely, at 42 h, no significant differences (*P* = 0.592) in Pi concentration was noticed between the PM and NM treatments (**Figure 3**). In both treatments, the relative Pi uptake/immobilization was 65% of the initial concentration of Pi in the bottles.

### Plant Growth Parameters and AMF-Root Colonization

At the end of the experiment, no significant differences were observed in SDW and RDW between the plantlets in the PM and NM treatments (**Table 1**). The height of plantlets in the PM and NM treatments were similar at the end of the experiment (i.e., 25 days – *P* = 0.14). For both treatments, a relative increase of 60% was noticed as compared to the initial height (PM plantlets 36.7 ± 1.2 cm and NM plantlets 32.4 ± 3.3 cm). Similarly, no significant differences were noticed in shoot P concentration and total P content of plantlets between the PM and NM treatment (**Table 1**). Conversely, the plantlets in the PM treatment had a significantly higher root P concentration and total P content as compared to the plantlets in the NM treatment (**Table 1**).

**Table 1:** Shoot and root dry weight (g), P concentration (mg g^-1^ of dry weight) and total P content (mg plant^-1^) of pre-mycorrhized (PM) and non-mycorrhized (NM) maize plantlets after 25 days of growth in perlite and after circulation through containers during 42 h of an 90% P-impoverished (i.e., *P*i = 6.245 mg L^-1^) Hoagland solution.

	Dry weight (g)		P concentration (mg g^-1^)		P content (mg plant^-1^)	
	Shoot	Root	Shoot	Root	Shoot	Root
PM	3.56 ± 0.08 a	1.55 ± 0.05 a	2.68 ± 0.07 a	1.8 ± 0.11 a	9.51 ± 0.19 a	2.72 ± 0.13 a
NM	3.47 ± 0.08 a	1.54 ± 0.06 a	2.85 ± 0.13 a	1.35 ± 0.04 b	9.86 ± 0.45 a	2.10 ± 0.11 b

*Data are mean values and standard errors (SE) of 9 (PM) and 8 (NM) replicates. Values within a column followed by different letters are significantly different (*P* ≤ 0.05, Student’s *t*-test).*

At the end of the experiment, the %TC, %AC, and %VC of the maize plantlets in the PM treatment was 90% ± 0.5, 64.5% ± 1.8, and 24.8% ± 1.4, respectively. Colonization before transfer to the containers was 80.7% ± 1.7, 35.9% ± 3.3, and 14.8% ± 1.5 for %TC, %AC, and %VC, respectively. The increase in colonization percentages was significant for %TC and %AC (*P* < 0.0001) and %VC (*P* < 0.005).

## Discussion

A non-destructive circulatory semi-hydroponic cultivation system with perlite as substrate was developed to investigate the short-term Pi depletion in a nutrient solution and by corollary Pi uptake/immobilization by non-mycorrhized and AMF-colonized maize plantlets. The system was adequate either for the maize plantlet and its fungal associate *R. irregularis*. Indeed, maize growth increased by 60% in a period of 25 days. Similarly, *R. irregularis* developed profusely with values of total root colonization reaching 90% in the same period. This suggested that the Hoagland^-P^ nutrient solution was adequate either to support plant growth and AMF development within the roots. These results are in agreement with Wright et al. (2005) and Tian et al. (2013), who demonstrated the ability of maize cultivars to be extensively colonized by the AMF *R. irregularis* at Pi concentrations below 1 mM.

Short-Term Pi uptake dynamics was first investigated by Colpaert et al. (1999) in roots of intact *P. sylvestris* seedlings colonized by four different ECM fungi and further extended to the same ECM-plant couples by Van Tichelen et al. (1999) to study Pi uptake under copper toxicity. For AMF, P acquisition by plants was mostly investigated using ^33^P or ^32^P (Smith et al., 2011; Walder et al., 2015; Kavka and Polle, 2016) or by destructive harvest of plants and analysis of P concentration in shoots and roots (Castillo et al., 2013; Nazeri et al., 2014; Xie et al., 2014; Gonçalves de Oliveira et al., 2017). For instance, Poss et al. (1985) analyzed P concentration from harvested plants as well as from the leachate volume collected during irrigation, to establish the P loss from mixed substrate. Hydroponic cultivation systems have also been used to determine P content in shoots and roots using different nutrient solutions (i.e., modified Long Ashton nutrient solution, modified Clark nutrient solution, half-strength Hoagland’s solution) (Ojala and Jarrell, 1980; Medeiros et al., 1994; Hawkins and George, 1997; White and Charvat, 1999). For instance, Ojala and Jarrell (1980) used a recirculating hydroponic sand culture system and demonstrated that at low concentration of Pi (i.e., 0.1 mg L^-1^) colonization by the AMF was higher, with numerous arbuscules and vesicles, as compared to a concentration of 0.3 mg P L^-1^. However, none of these studies considered depletion curves of Pi in a nutrient solution and by corollary Pi uptake/immobilization by intact plant-AMF associates. Thus, it seems that the circulatory semi-hydroponic system developed here could ease the determination of P dynamics in plant-AMF associations by the indirect analysis of Pi in the nutrient solution, avoiding disturbing the extraradical mycelium development, Pi uptake, translocation and transfer function and preventing to harvest plants.

Colonization of roots by *R. irregularis* resulted in a significant increase of Pi uptake in the PM maize plantlets as compared to the NM plantlets at 9 and 21 h, while no difference was noticed at 42 h in the experiments. The difference of Pi uptake at 9 and 21 h was probably not ascribed only to the higher volume of perlite explored by the AMF-colonized roots. Indeed, as suggested by Colpaert et al. (1999) using the same system with ECM fungi, the constant percolation of the nutrient solution during the uptake analyses counteracts the development of depletion zones around roots and hyphae. Therefore the high Pi-uptake rates of the mycorrhizal root systems cannot be ascribed uniquely to an improved nutrient scavenging of the substrate by the fungus but possibly also to a higher number of Pi transporters operating in the mycorrhizal root systems than in the NM roots (Colpaert et al., 1999). It is not excluded that these Pi transporters (on root and hyphae surface) were more abundant in the PM maize plantlets. Nevertheless, as reported by Tian et al. (2013), little is known about the relationship between the expression of Pi transporters in maize roots colonized by AMF and the growth or Pi uptake in maize. Indeed, even if short-term Pi uptake rates increased in the PM plantlets as compared to the NM ones, no difference in biomass was observed. This result corroborates the findings of Smith et al. (2004) in tomato. These authors suggested that there is no clear relationship between total root colonization and plant growth or P response.

The absence of differences in biomass between the AMF- colonized and control plantlets, could be attributed to the fact that each plant received strictly the same amount of nutrients flowing on the root system during several weeks and uptake rates is only established for short-term at fixed intervals and with refreshed nutrient solutions. In presence of AMF, the nutrient solution is much faster depleted, which would find its advantages in a non-resource restricted environment, thus impacting growth, while in the semi-hydroponic system, the same amount of nutrients would at the end be taken up in presence as well as in absence of AMF, resulting in the absence of visible impact on plant growth. This could possibly explain the absence of difference at 42 h for the experiments.

The higher P concentration and content in the root system of the PM plantlets, suggested a better P nutrition of the PM plantlets. Curiously, these differences were not reflected in the shoot parts. This could indicate that P was accumulated in the intraradical hyphae of the AMF after synthesis into polyphosphate granules (poly-P) in the vacuole of the extraradical/intraradical hyphae (Ezawa et al., 2001; Zhang et al., 2016). Fiorilli et al. (2013) suggested that, once the AMF satisfied their Pi requirements, most of the Pi flux is oriented toward the host. Because of the high presence of the fungus inside the roots (90% of colonization rate), this stored poly-P could represent a reservoir of P available for the fungus metabolism or for plant growth. Nazeri et al. (2014) also observed that after the P pulse, shoot P concentration increased by 143% in uncolonized plants, while in colonized plants the increase was only 53% resulting in shoot P being highest in uncolonized plants. They suggest that AMF affects shoot P concentration at low and high availability, restricting its transfer when P becomes excessive to plant requirements.

The major advantage offered by the circulatory semi-hydroponic cultivation system developed in the present study is the capacity to monitor and compare non-destructively the Pi uptake dynamics of mycorrhizal and non-mycorrhizal plants via the depletion of Pi in the nutrient solution flowing through the plantlets containers. The system could be extended to analyze the uptake dynamics of other macro and micro-nutrients from their depletion in a nutrient solution, as well as to the dynamics of uptake of various plant species at different phenological development steps (e.g., at vegetative growth, flowering, production of seeds, senescence) associated to AMF having different life history strategies and under non-stress as well as abiotic (e.g., salinity, pollution by hydrocarbons pollutants or potential toxic elements) stress conditions. However, complementary studies requiring an improvement of the circulatory semi-hydroponic cultivation system are necessary to separate the contribution of roots and fungi to the uptake, and thus to determine the relative contribution of each in the P uptake dynamics and accumulation within plants.

## Author Contributions

MG-R: Data collection, analysis and interpretation, drafting the work, commentaries corrections, final approval, and agreement with all aspects of the work. MC-S: Data collection, analysis and interpretation, draft work commentaries and corrections, final approval and agreement with all aspects of the work. KP: Development of experiment, data collection, final approval and agreement with all aspects of the work. CM: Development of the system and preliminary experiment, final approval and agreement with all aspects of the work. MN-M: Development of the system and experiment, data collection, data analysis, interpretation of data from preliminary experiment, final approval and agreement with all aspects of the work. SC: Contribution to the development of the experiment, draft correction and final approval and agreement with all aspects of the work. SD: Substantial contributions to the conception and design of the experiments, interpretation of the data, draft corrections final approval and agreement with all aspects of the work.

## Conflict of Interest Statement

The authors declare that the research was conducted in the absence of any commercial or financial relationships that could be construed as a potential conflict of interest.

## Footnotes

1 http://www.mycorrhiza.be/ginco-bel

2 http://www.cipf.be/
